# Utilization of Whole Exome Sequencing in Lethal Form of Multiple Pterygium Syndrome: Identification of Mutations in Embryonal Subunit of Acetylcholine Receptor

**DOI:** 10.22088/IJMCM.BUMS.8.4.258

**Published:** 2019

**Authors:** Tahere Nazari, Ali Rashidi-Nezhad, Maziar Ganji, Zahra Rezaei, Saeed Talebi, Nasrin Ghasemi, Javad Tavakkoly Bazzaz

**Affiliations:** 1 *Department of Medical Genetics, School of Medicine, Tehran University of Medical Sciences, Tehran, Iran.*; 2 *Maternal, Fetal and Neonatal Research Center, Tehran University of Medical Sciences, Tehran, Iran.*; 3 *Department of Medical Genetics, School of Medicine, Shahid Beheshti University of Medical Sciences, Tehran, Iran.*; 4 *Department of Medical Genetics, School of Medicine, Iran University of Medical Sciences, Tehran, Iran.*; 5 *Abortion Research Centre, Reproductive Sciences Institute, Shahid Sadoughi University of Medical Sciences, Yazd, Iran.*

**Keywords:** Multiple pterygium syndromes, whole-exome sequencing, CHRNG, recurrent abortion

## Abstract

The acetylcholine receptor (AChR) is a member of the superfamily of transmitter-gated ion channels having a critical role in controlling electrical signals between nerves and muscle cells. Disruptive mutations in genes encoding different subunits of AChR result in multiple pterygium syndrome (MPS), which can be associated with a severe prenatally lethal presentation. This study aimed to investigate the etiology of lethal MPS (LMPS) in two consanguineous families with a history of miscarriages. DNA was extracted from a tissue sample of two aborted fetuses (probands) from two different families with a history of spontaneous miscarriages. Parental peripheral blood samples were collected for confirmatory analysis and follow-up testing. Whole-exome sequencing (WES) was performed on DNA from the probands. The results were confirmed and segregated by Sanger sequencing. Moreover, protein structure evaluations were accomplished. We identified a homozygous frameshift mutation of c.753_754delCT (p.V253fs*44) and a homozygous missense mutation of c.715C>T (p.Arg239Cys) in the *CHRNG* gene. Both aborted fetuses had pterygium, severe arthrogryposis, and fetal hydrops with cystic hygroma, being compatible with LMPS. The heterozygous state was confirmed in parents for both *CHRNG *variants. Likewise, *CHRNG* mutation was predicted to display the damaging effects by lowering the number of helixes and modifying the surface electrostatic potential. The present study identified rare sequence variants in the *CHRNG* gene in aborted fetuses from consanguineous couples with recurrent miscarriage history. WES is a comprehensive and cost-effective approach to study heterogeneous diseases including MPS. Such findings can improve our knowledge of MPS databases, particularly for genetic counseling of high-risk families and preimplantation genetic diagnosis.

Multiple pterygium syndrome (MPS) comprises a group of multiple congenital anomaly disorders that are clinically and genetically heterogeneous(1, 2). The non-lethal variant of arthrogryposis multiplex congenita is defined by excessive webbing (pterygia), congenital contract-ures (arthrogryposis), scoliosis, and other possible features (3). However, the lethal form of this disorder (LMPS, OMIM 253290) is considered to be fetal before birth or very soon after birth. Lethal MPS follows autosomal and X-linked recessive inheritance pattern and is an extremely rare genetic condition affecting the skin, muscles, and skeleton and is commonly accompanied by minor facial abnormalities, prenatal growth deficiency, spine defects, joint contractures, and pterygia of the neck, elbows, back of the knees, armpits, and fingers. Fetuses with this condition are usually not born. Although autosomal recessive inheritance appears to be the most common form of this disease (3, 4), X-linked and autosomal dominant inheritance patterns are, also, observed in a few cases (5).

A huge body of literature represents that mutations in the cholinergic receptor nicotinic gamma subunit (*CHRNG*) gene lead to autosomal recessive MPS (6).The acetylcholine receptor (AChR), playing role as an excitatory cation channel, is a member of the superfamily of transmitter-gated ion channels with a critical role in controlling electrical signals between nerves and skeletal muscle cells acting through opening and closing a membrane-spanning pore (7). In humans, AChR is a transmembrane pentameric glycoprotein having four different types of subunits including two α (*CHRNA1*), one β (*CHRNB*), one δ (*CHRND*), and one γ/ε (*CHRNG*/*CHRNE*) subunits. Interestingly, the AChR is available in two forms namely embryonic, which is present in fetal and denervated muscle, and the adult, predominantly expressed after birth. At the 33^rd^week of gestation, the embryonic AChR is switched to the adult type (8, 9). Worthy of note, the *CHRNG* gene, encoding the γ subunit of AChR, is merely expressed during early fetal development, while it is replaced by *CHRNE*, encoding ε subunit, in adult humans. Mutations in the *CHRNG* gene can result in both non-lethal Escobar variant-MPS (EVMPS) and lethal MPS (LMPS) (10).

Whole-exome sequencing (WES) has been successfully used, as an edge-cutting approach, to study the protein-coding and significant flanking regions of the genome contributing to more than 85% of the monogenic disorders. This high-throughput technique helps to reveal the disease-causing variants with low cost and high resolution in comparison with other diagnostic molecular methods such as single locus-based and panel-based sequencings in a wide range of inherited diseases. So far, it has been used in several medical areas particularly in consanguineous families with recurrent miscarriage to identify the precise etiology (11).

Altogether, given the fact that a few studies have been so far conducted on molecular etiology of MPS in Iranian population, our goal here was to perform a WES on the aborted fetus resulted from a consanguineous healthy parents of Iranian Cauc-asian descent with two previous miscarriages. These results were confirmed and segregated via Sanger sequencing in the fetus and parents. Furthermore, protein structure evaluation shed more light on the possible underlying mechanisms of the obtained mutation pathogenicity.

## Materials and methods

**The patients**

In this study, two patients from two different familis were studied. Both couples were **referred to the Yas Women's General Hospital, affiliated with Tehran University of Medical Sciences, for genetic counseling. Written informed consent was obtained from all individuals. The whole protocol of this study was approved by the Ethics Committee of the Tehran University of Medical Sciences (with ethics ID of IR. TUMS. MEDICINE. REC. 1398.193).**

**Patient 1**

**Fig. 1:**
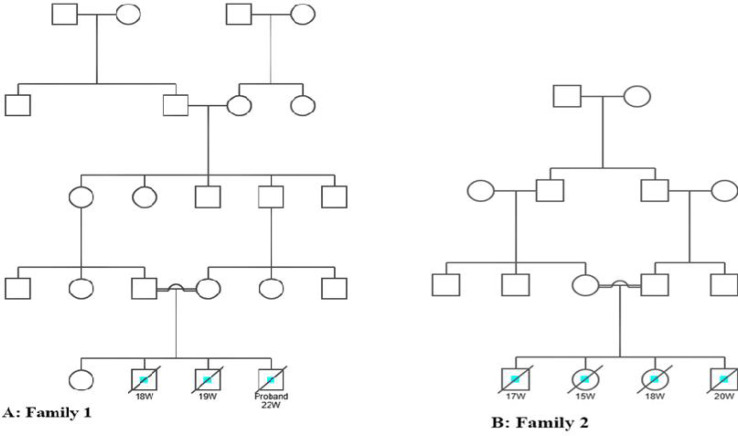
**Pedigrees for families described in this study.**Affected individuals are represented with shaded symbols

A tissue sample was collected from the aborted fetus (proband) in family A. Although the mother had history of two spontaneous miscarriages at 18 and 19 weeks of gestation, no abnormalities were found regarding medical history, physical and laboratory investigations (Figure 1A). The fetus had short neck, hydrothorax, cystic hygroma, and kyphosis, as well as short trunk because of scoliosis. At 22 weeks of gestation, the pregnancy was terminated and the post mortem examination did not reveal any sign of intrauterine growth retardation; nevertheless, craniofacial anomalies, including hypertelorism, low set ears, downslanting palpebral fissures, and high arched palate, were seen. There was evidence of flexion contractures in shoulders, elbows, knees, and ankles in addition to the webbing of elbows, axillae, and groins.

**Patient 2**

The parents in the second family (called family B in this study) were first cousins and had the history of three spontaneous miscarriages at 17, 15, and 18 weeks of gestation (Figure 1B). Both mother and father were apparently normal in terms of pregnancy and other related laboratory tests. For this family, the pregnancy loss at 20 gestation weeks was determined. Phenotypes were abnormalities in skin, muscles, and skeleton exhibited in the aborted fetus. Plus, minor facial abnormalities, spine defects, joint contractures, and pterygia of the neck, elbows, back of the knees, armpits, and fingers were noted.

**DNA extraction and WES pipeline**

Parental peripheral blood samples were collected in EDTA tubes for confirmatory analysis and follow-up testing using DNA extraction kit (QIAGEN, Hilden, Germany). The purity and quality of DNA samples were investigated by Thermo Scientific™ NanoDrop™ One Microv-olume UV-Vis spectrophotometer (Thermo Scientific, Waltham, MA, USA). The optical density of DNA samples was between 1.8 to 2.0. Moreover, we examined the DNA integrity through agarose gel-electrophoresis.

The WES was used on DNA from the proband. As the next step, DNA was fragmented and the targeted enrichment was done using the SureSelect Human All Exon V6 kit (Agilent Technologies, Santa Clara, CA, USA). Libraries were sequenced on the Illumine HiSeq 2000 platform for 2*100 bp reads with the average coverage of 60X(Eurofins Genomic Services, India). The sequence read quality assessment was done by generating quality control (QC) with FastQC. Burrows-Wheeler Alignment (BWA) algorithm was used with default parameters for alignment to (human) genome assembly GRCh37 (hg19). Picard-Tools was used to mark and remove the duplications, and then variant calling was performed with HaplotypeCaller algorithm in Genome Analysis Toolkitversion 4.0 (GATK4) package. 

Variants were annotated with ANNOVAR using various public and in-house databases.The allele frequency was searched in 1000 Genomes Project (approximately 6500 exomes from the NHLBI), exome sequencing project (http:// esp.gs. washington.edu), the exome aggregation conso-rtium (ExAC, http:// exac.broadinstitute.org/, providing exome sequencing data from several large-scale sequencing projects), genome aggre-gation database (gnomAD, https:// gnomad. broadinstitute.org/), and Iranome( http:// www. iranome.ir/gene/ ENSG00000196811, to exclude ethnic- specific variants). Furthermore, the functional analysis tools, MutationTaster, as well as, genomic evolutionary rate profiling (GERP) scores were used in combination for functional effect prediction. The possible damaging effect of the variant on function and structure was estimated by SIFT (https:// sift. bii. a-star.edu.sg/) and PolyPhen2 (http:// genetics. bwh. harvard. edu/ pph 2/).

Being aware of consanguinity in this family and the autosomal recessive nature of the disorder, as well as its conservation across species, the filtration strategy was focused on minor allele frequency less than 1 percent (MAF<1%), and homozygous exonic variants (including missense, splice site, start and stop codon, and nonsense) were predicted to be damaging. The WES results were validated by Sanger sequencing to verify whether the identified potentially disease-causing variants were true variants or sequencing artifacts. Sanger sequencing for the obtained variants was performed using gene-specific primers designed by Allele ID primer design software version 7 (Premier Biosoft, Palo Alto, USA). Primer sequences will be available on request.

**Protein structure predictions**

**Prediction of secondary structures**

The amino acid sequence of acetylcholine receptor subunit gamma (ACHG) in human (P07510)was obtained from UniProt databank (http://www.uniprot.org/). Secondary structures of the native and mutant proteins were predicted by PHD (https://npsa-prabi.ibcp.fr/), which is an automatic mail server for protein secondary structure prediction (12, 13). 

**Tertiary structure prediction and validation**

The three-dimensional structural predictions of ACHG and its mutant protein were generated using the iterative threading assembly refinement (I-TASSER) server (14). The top ranking models were refined by GalaxyRefine server (15). The quantitative evaluation of selected models were carried out using VADAR and ProSA web tools (16, 17).

**Visual presentation**

All protein figures were created using PyMOL software (18).

## Results

**WES findings**

A homozygous frameshift mutation of c.753_754delCT (p.V253fs*44) and a homozygous missense mutation of c.715C>T (p.R239C), both in *CHRNG* gene (NM_005199.4), were identified by WES in aborted fetuses of family A and family B, respectively. These female (in family A) and male (in family B) fetuses were born from healthy consanguineous, first cousin, parents with Iranian descent (Figure 1).

According to the American College of Medical Genetics and Genomics (ACMG) guidelines, all observations provided enough evidence of patho-genicity for the above-mentioned *CHRNG* mutation and helped us to classify it as a pathogenic variant. Moreover, multiple lines of computational evidence supported a deleterious effect on the gene/gene product. According to the result of bioinformatics prediction tools, the c.753_ 754 delCT, (p.V253fs*44) variant in exon seven of *CHRNG* gene consisted with deleterious effect, and it can be categorized as a disease-causing variant by SIFT, PolyPhen2, Provean, MutationTaster, VEP, and GERP in addition to ClinVar (Table 1). In the fetus of family B, the detected mutation (c.715C>T, p.Arg239Cys) was categorized as a pathogenic variant based on ACMG guidelines criteria*. In silico* prediction methods, including SIFT, PolyPhen2, Provean, MutationTaster, and GERP in addition to ClinVar, also suggested a deleterious effects for this variant.

**Fig. 2:**
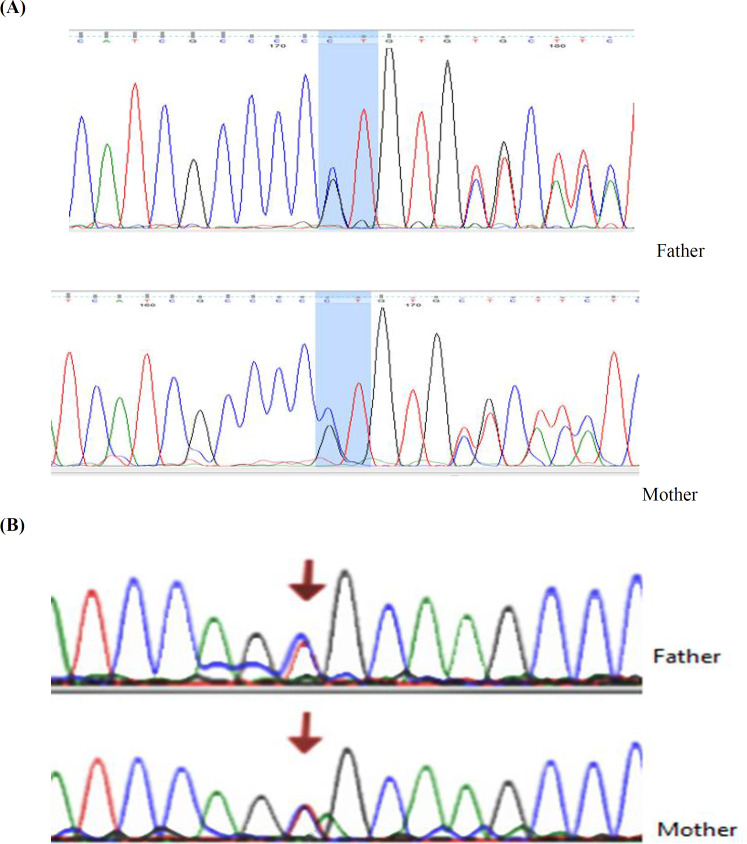
**Chromatograms of** ***CHRNG*** **mutations.**(A) c.753_754delCT and (B) c.715C>T variants in *CHRNG* gene in parents of both families. The Sanger sequencing results demonstrated the heterozygous status in father and mother of both fetuses

**Fig. 3:**
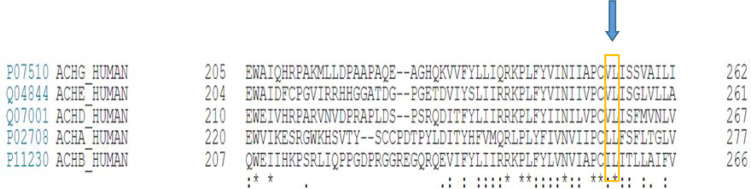
Evolutionary conservation of residue V253 relevant for mutation c.753_754delCT (p.V253fs*44).

In addition, Sanger sequencing chromatograms indicated the homozygous status of two observed mutations in aborted fetuses, while the heterozygous status was observed in parents in both families (Figure 2).The position of these variants is highly conserved in human β, δ, γ, and ɛ subunits,as well as among γ subunits within species analyzed (Figure 3).

**Prediction of secondary and tertiary structures in addition to structural validity**

PHD online server was used for prediction of the secondary structure of native and mutant ACHG protein. As shown in Table 2, the most second structures were random coils for both native and mutant protein; however, helixes are more predicted in native structure. 

**Table 1:** The results obtained from different population and *in silico* prediction databases for p.V253fs*44 and p.Arg239Cys variants in *CHRNG* gene

**CHRNG gene variants**	p.V253fs*44	**p.Arg239Cys**
MAF in TOPMED	0.00034 (43/125568)	0.00001 (1/125568)
MAF in ExAC	0.00018 (22/121412)	0.00001 (1/121410)
MAF in GnomAD EXOM	0.00016 (41/251490)	T=0.00002 (4/251486)
MAF in GnomAD)	0.0002 (6/31398)	-
MAF in ESP	0.0018 (22/12520)	-
MAF in Estonian	0.001 (3/4480)	-
MAF in GO-ESP	0.0018 (22/12520)	-
MAF in ALSPAC	0.001 (2/3854,)	-
MAF in PAGE_STUDY	0.0003 (26/78694)	0.0000 (1/78654)
MAF in Estonian	0.001 (3/4480)	-
MAF in TWINK	0.000 (0/3708	-
MAF in Iranome	0	0
SIFT	0	0
PolyPhen2	0.08	1
Provean	1	1
MutationTaster	Disease-causing	Disease causing automatic
PPI	9	
GERP NR	5.1799	5.1799
VEP	High impact	MODERATE
ClinVar	Pathogenic	Pathogenic

Residue V253 is completely conserved in all human subunits a (CHRNA1), b (CHRNB1), d (CHRND), e (CHRNE), and g (CHRNG).

**Table 2:** Secondary structural elements of native and truncated proteins

**Structure**	**α-helices (%)**	**Random coils (%)**	**Extended strand (%)**
**Native structure**	29.2	49.3	21.5
**Truncated structure**	7.0	55.7	37.4

**Table 3:** The Ramachandran results retrieved by VADAR web server

**Protein**	**Residue in phipsi ** **core**	**Residue in phipsi allowed**	**Residue in phipsi generous**	**Residue in phipsi outside**
**Native protein**	83%	11%	2%	1%
**Truncated protein**	83%	12%	1%	2%

The five predicted models of tertiary structure of the native and mutant protein were obtained by I-TASSER server and the best models were model 1 with the best C-score. The C-score, TM score and RMSD of native and mutant proteins were -1.24, 0.56±0.15, 10.3±4.6Å and 0.40, 0.77±0.10, 5.2±3.3Å, respectively. The analysis of predicted 3D model of proteins was validated using VADAR and ProSA web servers. The Ramachandran result analsyses of proteins by VADAR are shown in Table 3. As well, ProSa server displayed that the Z-scores of native and mutant proteins were -5.94 and -3.88, respectively. For visualization, the mutant model was superimposed with native model using PyMOL software (Figure 7A). Furthermore, surface electrostatic potential of native and mutant proteins was calculated by PYMOL software (Figure 7B and C). This calculation uncovered that mutant protein carries more negative charge (-14) than native protein (-7).

## Discussion

In the present study, we performed WES on two aborted fetuses from Iranian consanguineous families with history of recurrent miscarriage, and interestingly identified a homozygous mutation of c.753_754delCT (p.V253fs*44) in *CHRNG* gene in the fetus of one family and a homozygous missense mutation of c.715C>T (p.R239C) in *CHRNG* gene in that of other family, being confirmed for heterozygous status in parents of both families. As shown in Table 1, all *in silico* tools used in this study suggested a deleterious role for these variants. Likewise, their minor allele frequency (MAF) in almost all population databases, including but not limited to ExAC, GnomAD, and ESP, was less than 0.001 percent, while not being reported in Iranome database. Moreover, based on computational data, this mutation is located in an evolutionarily highly conserved region. *CHRNG* mutations and sequence variants that have been reported in ClinVar database and previous investigations are described in Table 4 and Figure7.

AChR is a member of transmitter gated ion channel superfamily. Two forms of skeletal muscular AChR are found in mammalian and identified by their different function and subunit compositions (19). Gamma subunit is expressed in fetus and epsilon is expressed in mature skeleton. Fetal AChR type is present before week 33 of gestation in human, and is gradually replaced with the epsilon subunit mediated by acetylcholine receptor-inducing activity (ARIA) (20-22). Before the insertion of receptor into the membrane, the five subunits of AChR should be assembled in the endoplasmic reticulum (ER) (23, 24). The interaction network of *CHRNG*, *CHRNA1*, and *CHRND* with associated genes in MPS is shown in Figure 7. The receptor cannot reach the cell surface whether one subunit is missing or due to AChR deficiency (25). It was previously reported that disruptive mutations in different genes encoding subunits including *CHRNG*, that interfere with the correct localization of the receptor in the cell membrane, have been associated with MPS. Traditionally, MPS is divided into a mild form of nonlethal (Escobar) type (OMIM 265000) and the severest form, prenatally lethal (OMIM 253290). The gamma subunit contributes to neuromuscular signal transduction and also neuromuscular organogenesis (25). Therefore, *CHRNG* gene is regarded as essential in human for early develo-pment. Hoffmannet al. (2006) formerly indicated the lack of fetal AChR at the cell surface in absence of the γ subunit*in vitro* experiments (25, 26). Inappropriate AChR function in fetal life results in reduced prenatal muscle strength and movement (27).

**Table 4:** The *CHRNG* mutations and sequence variantsrelated to MPS disease reported in previous studies and ClinVar database

**Nucleotide alterations**	**Alterations in the coding sequence**
c.13C>T	(p.Gln5Ter)
c.117dupC	(p.Asn40GlnfsTer96)
c.136C>T	(p.Arg46Ter)
c.202C>A	(p.Arg68=)
c.301_309dupAGGGTGCCG	(p.Arg101_Pro103dup)
c.320T>G	(p.Val107Gly)
c.397delT	(p.Ser133ProfsTer50)
c.401_402delCT	(p.Pro134ArgfsTer43)
c.428C>G	(p.Pro143Arg)
c.459dupA	(p.Val154SerfsTer24)
c.715C>T	(p.Arg239Cys)
c.753_754delCT	(p.Val253AlafsTer44)
c.1408C>T	(p.Arg470Ter)
c.1210C>T	(p.Gln404Ter)
c.301_309dup	(p.Pro103_Ser104insArgValPro)
c.274C>T	(p.Arg92Ter)
c.1292_1311del20	(p.Leu431HisfsX22)
c.388delG	(p.Val130CysfsX53)
c.1132_1136dup	(p.Gly380ProfsX39)
c.55G→A	(p.Gly19Arg)

**Fig. 4:**
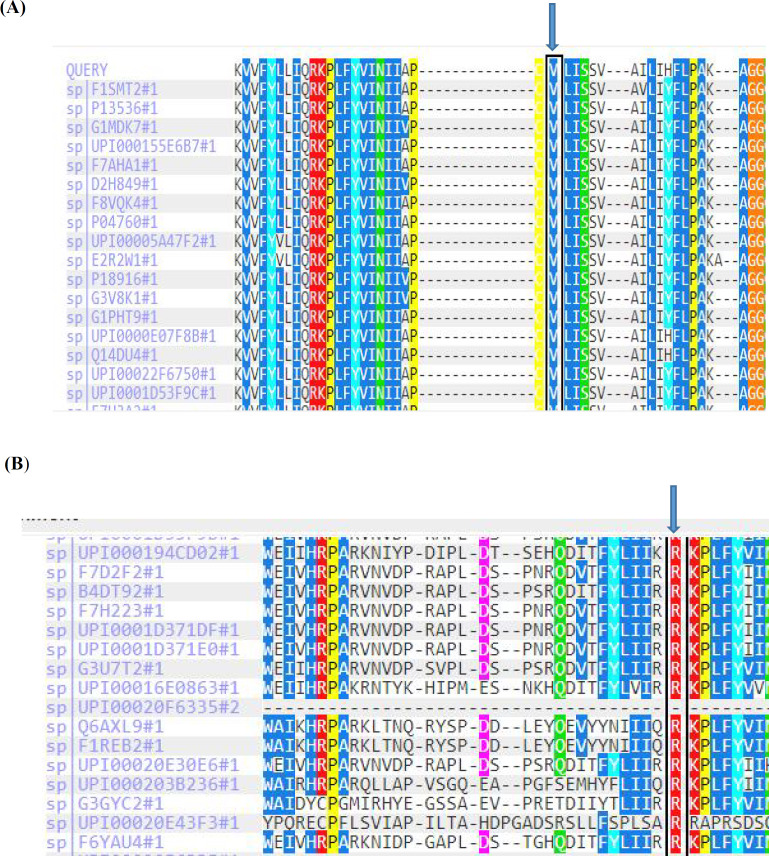
Multispecies alignment for the candidate variants of c.753_754delCT [p.V253fs*44] (A) and c.715C>T [p.Arg239Cys] (B) within all species analyzed.Arrows indicate the mutations found in the present study

In line, considering that many aspects of embryonic development are conserved among mammals, it is very likely that the critical genes in this pathway may be significantly similar to that of human embryos, offering mice phenotype information as a valuable tool for gene prioritization of prenatal lethal phenotypes. Both valine and arginine residues are conserved in human β, δ, γ, and ɛ subunits, as well as among vertebrate γ subunits (Figure 3). Multispecies alignment for the candidate variants of c.753_ 754delCT (p.V253fs*44)and c.715C>T (p.R239C) indicated high conservation within all species analyzed (Figure 4).*In vivo* study on mice with the autosomal recessive mutation has shown a similar phenotype- genotype correlation that displays the key role of the AChR gene subunits in the neuromuscular development pathway. Lack of γ subunit in mice caused muscular weakness, hind limb paralysis, feeding problems, stillbirth, and death within 48 h due to respiratory failure (28). 

Both families studied here had background of unexplained miscarriages. Known risk factors of recurrent miscarriages such as abnormal couple and fetus karyotypes, antiphospholipid and lupus antibodies, prothrombotic and endocrine elements, hormonal levels, and uterine anatomy alteration were all excluded. We hypothesized that given the likely autosome recessive nature of the disorder and consanguinity in these families, there should be a priority for variants with homozygous and compound heterozygous inheritance patterns in filtering step. We identified c.753_754delCT and c.715C>Tmutations in *CHRNG* gene in fetuses with a lethal form of MPS from consanguineous couples with previous miscarriages. As exhibited in Figure 7 and Tables 2 & 4, further bioinformatics analysis by investigating secondary and tertiary protein structures predicted that, by lowering the number of helixes and modifying the surface electrostatic potential, this variant was pathogenic and can be strongly associated with embryonic lethality. Kariminejadet al. used WES to study 9 Iranian cases, 2 fetuses and 7 children, with a clinical presentation of arthrogryposis in three families with EVMPS and reported one novel and two previously reported frameshift mutations in *CHRNG*(10). Moreover, the homozygous c.753-754delCT (pVal253Alafs44Ter) mutation was found in cases with the lethal and non-lethal MPS, born to a consanguineous healthy couple of Turkish origin (29, 30). 

**Fig. 5:**
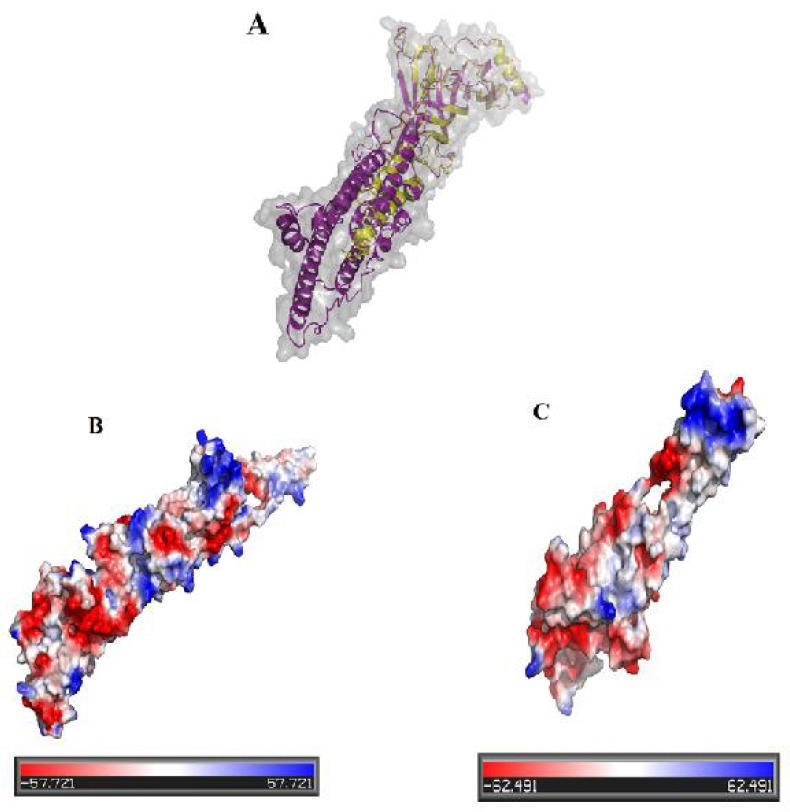
**Predicted structure of native and truncated protein.** (A) Superimposition of the best predicted models of native and truncated protein.The native protein structure was shown in magenta and truncated protein in yellow. Surface electrostatic potential of native protein (B) and mutant proteins (C);blue, red and white colors represent the positive, negative and hydrophobic regions, respectively

**Fig. 6:**
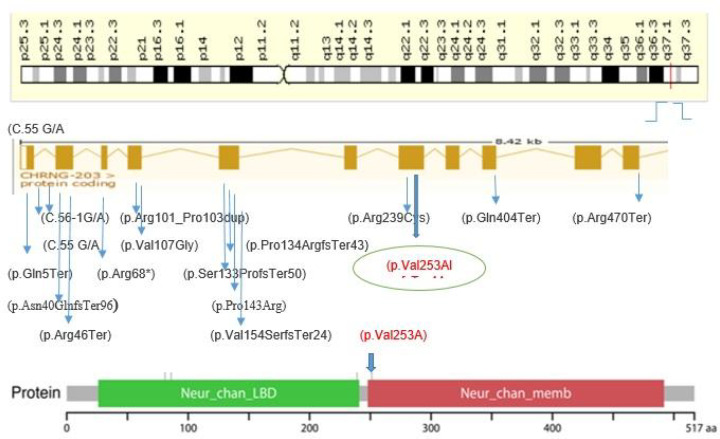
Schematic representation for localization of the identified *CHRNG* variants in neurotransmitter-gated ion-channel transmembrane region

**Fig. 7:**
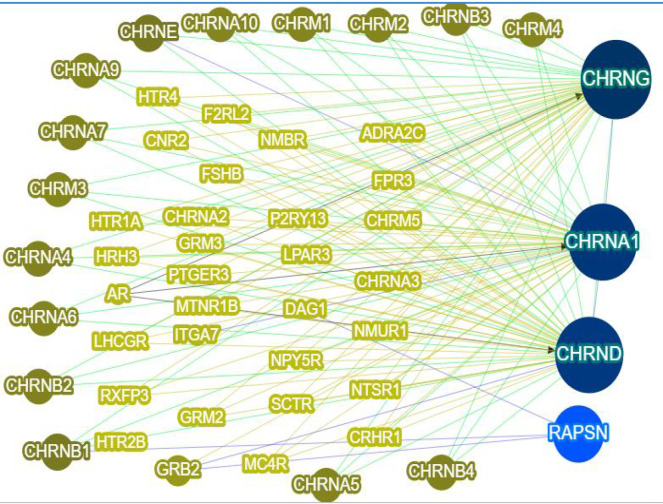
**The interaction network of** ***CHRNG*** **with other genes, such as** ***CHRNA1*** **,** ***CHAND*** **, and** ***RAPSN*** **(dark blue circles).** Gray circles and green rectanglesindicate the other genes in the same network but not associated with MPS (Gene-gene interactions received from http://phenolyzer.usc.edu/).

Vogt et al. studied one hundred families with at least one affected individual with clinical features of EVMPS, LMPS or FAD and 20 *CHRNG* mutations were detected. The c.459dupA (p.Val154SerfsX24) and c.753_754delCT (p.Val253AlafsX44) mutations, as the most frequently observed *CHRNG* alterations, were identiﬁed from different ethnic backgrounds affected with both EVMPS and LMPS in the homozygous state and with a second *CHRNG* mutation in two compound heterozygous individuals (30). There are similarities between the attitudes expressed by Kariminejad team and those described by Vogt et al. (2012) and Thompson et al. (1987). The evidence from these studies suggests that there is no apparent difference in mutation spectrum between EVMPS and LMPS cases neither the mutation type nor its position in the gene appeared to correlate with the severity of the phenotypes (30, 31). The *CHRNG* nonsense mutations, including c.753-754delCT, may cause both lethal and non-lethal MPS. Surprisingly, even severity of the phenotype varies significantly both within inter- and intra-families with the same mutation (29-31). In addition, the second *CHRNG* mutation, c.715C>T, was observed in MPS patients from unrelated families with Lebanon and Turkey origins, which recommends the close distribution of this mutation in Middle East region .The clinical phenotypes of these patients included contractures of elbow and knees, short neck, small and low set ears, micrognathia, and down-slanting palpebral fissures, being similar to that of our patients.Gene-gene interaction may be considered as a possible cause for this phenotypic variability besides other factors such as genetic background and inter-individual differences, and environmental modifiers. However, little is known about such interactions and there is an urgent need for establishing a greater degree of accuracy in the selection of candidate gene and also possibly related pathways of MPS. **In order to overcome the limitations of our study, one can include larger sample size of Iranian patients, in addition to performing the functional assays, to investigate other novel variants and their principal molecular mechanism in this disorder as the future steps.**

In conclusion, we reported c.753_754delCT and c.715C>T mutations in *CHRNG* gene in aborted fetuses as a possible contributing factor for MPS in two Iranian families with background of previous miscarriages. Such findings can improve our knowledge of genetic databases of patients with MPS, particularly forgenetic counseling of high-risk families and preimplantation genetic diagnosis. Further investigations are required to shed more light on the underlying mechanisms and the *CHRNG* function in human development.
